# The extremely rare case of aortic chordae tendineae strands: A case report with literature review

**DOI:** 10.1002/ccr3.8033

**Published:** 2023-10-10

**Authors:** Nakisa Khansari, Amir Mohammad Salehi

**Affiliations:** ^1^ Department of Cardiology, School of Medicine Hamadan University of Medical Sciences Hamadan Iran; ^2^ Student Research Committee, Hamadan University of Medical Sciences School of Medicine Hamadan Iran

**Keywords:** abnormal fibrous strand, aortic chordae tendineae strands, aortic regurgitation, aortic valve, echocardiography

## Abstract

The aortic chordae tendineae strands (ACTS) is a rare complication that can induce aortic regurgitation. Reported cases of ACTS are very few, and this is the first case reported in Iran. Patients with unexplained aortic regurgitation should be carefully evaluated for ACTS, which can be easily observed by TEE; a decision regarding aortic valve surgery should be made based on the severity of AR. Herein we reported A 64‐year‐old male was admitted to our hospital for dyspnea on exertion. In transthoracic Echocardiography a fibrous band‐like chordae in the aortic root attached to the noncoronary cusp of the aorta was seen, which caused retraction of the noncoronary cusp, mal‐coaptation of the aortic valves, and severe eccentric jet posterior directed aortic regurgitation. As a result of the ACTS, the patient was diagnosed with severe aortic regurgitation (AR); due to the symptomatic severe AR, the patient underwent aortic valve surgical replacement.

## Graphical abstract

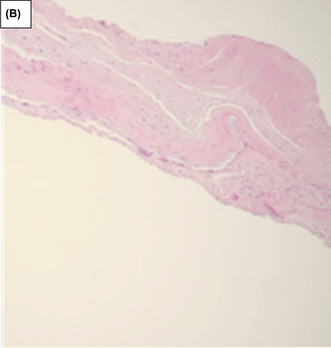

## 1 INTRODUCTION

The aortic chordae tendineae strands (ACTS), also known as “fibrous strands” in the literature, are believed to be embryonic remnants of the process of cusp formation. Rarely have ACTS on bicuspid and tricuspid aortic valves (AV) caused spontaneous aortic regurgitation (AR).^1^, ^2^ In this study, we present the case of a patient with tricuspid AV with normal thickening and ACTS. The patient had a smoking history and suffered from dyspnea and severe AR due to ACTS attached to a noncoronary cusp (NCC).

## 2 CASE REPORT

A 64‐year‐old male was admitted to Farshchian Cardiovascular Hospital for dyspnea on exertion (functional class II). The patient reported a smoking history but had no cardiovascular disease history. Physical examination revealed a systolic murmur III/VI and early to mid‐diastolic murmur in the right parasternal. The individuals' electrocardiogram and lung sounds were normal. No fever or chills were observed, and laboratory findings (complete blood count, serum inflammatory markers, and blood cultures samples) were within normal range. On the chest x‐ray, the trachea was observed in the midline, the cardiothoracic ratio was normal, the ascending aorta was dilated, and there were mild prominent bilateral hila and pulmonary vascular markings.

**FIGURE 1:**
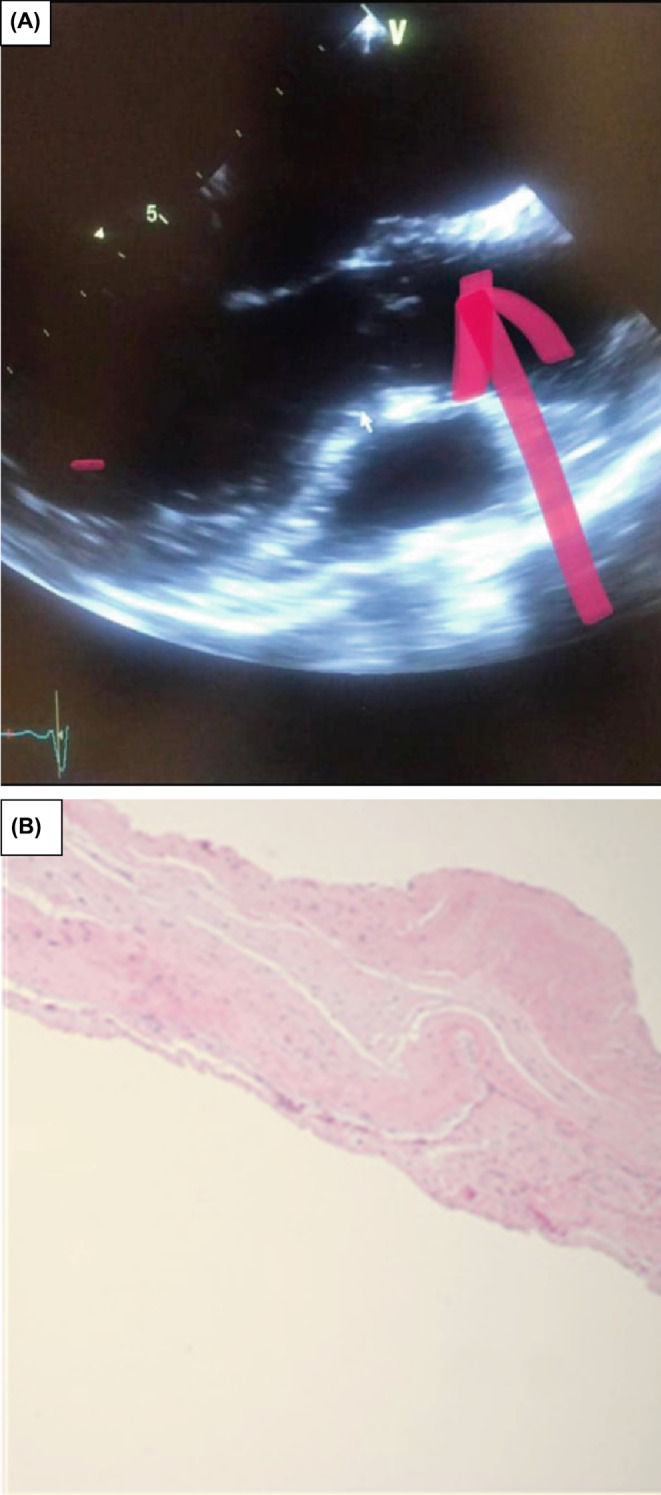
(A) ACTS (pink arrow) attached to the NCC. (B) Pathological examination showed fibrous tissue of AV.

Coronary and Aortic Computed tomography angiography (CTA) was also performed and revealed patent coronary arteries and normal‐sized ascending aorta, root, and descending aorta without intimal flap and complex plaque. Furthermore, transthoracic (TTE) and transesophageal (TEE) echocardiography performed in our hospital indicated dilatation of the aortic root (diameter at the sinus of Valsalva: 44 mm) and ascending aorta (aortic annulus 24 mm, ascending aorta 40 mm), mild left ventricle enlargement (Left‐Ventricular‐End‐Systolic‐Volume:60CC/m^2^) with mild systolic dysfunction, (LVEF: 45%–50%), and normal right ventricle size and systolic function. No significant abnormality was observed on the mitral valve except for mild mitral regurgitation. Moreover, AV were tricuspid with normal thickening; however, a fibrous band‐like chordae in the aortic root attached to the NCC of the aorta caused retraction of the NCC, malcoaptation of the AV, and a severe eccentric jet posteriorly directed AR (Figure 1A, and supplementary file). Mild pulmonary arterial hypertension (systolic pulmonary pressure: 40 mmHg, tricuspid regurgitation peak gradient (TRPG) was 35 mmHg and right atrial pressure was 5 mmHg) was also detected.

Consequently, the patient was diagnosed with severe AR due to the ACTS, and AV surgical replacement was performed due to the symptomatic severe AR. The aortic annulus was normal, the left ventricle was enlarged, and the ACTS was attached to NCC during the intraoperative examination (Figure 1B), a mechanical valve was implanted and the patient was discharged in good condition after 4 days, with a prescription for warfarin.

## 3 DISCUSSION AND CONCLUSION

AR caused by the ACTS is rare; it was previously believed to be associated with bicuspid AV and infrequently with tricuspid AV^3^; however, several reports have recently documented tricuspid AV cases.^4^ Most ACTS in the right coronary cusps or NCC, although patterns of ACTS attachment varied in each case^5^; however, most patients with ACTS have been from Japan and other Asian countries, indicating that heredity may play a role in the formation of ACTS.^4^ ACTS was coupled to NCC in our patient.

If the ACTS efficiently maintains balance, no valve dysfunction with severe regurgitation will develop; however, two mechanisms have been proposed to cause AR in patients with ACTS: first, the ACTS rupture between the aortic cusp and aortic wall, and second, the ACTS' restricted closure of the AV.^1^, ^6^ Our case may be an example of the second mechanism's involvement; however, most reported cases are associated with the first mechanism.

Patients with unexplained AR should undergo a thorough evaluation for ACTS, which TEE more easily observes than TTE.^1^ Additionally, three‐dimensional TEE plays a significant role in making a diagnosis.^7^

In our patient both TTE and TEE echocardiograms were performed, and both revealed abnormal lines in the aortic valve. ACTS should be distinguished from Lambl's excrescences and infectious and noninfectious neoplasms when abnormal lines are detected at the AV.

Thin, filiform, or papillary structures called Lambl's excrescences can be found on the outer edges of valves in elderly individuals. These structures are more commonly seen on the MV near its closure line, rather than on the AV. Despite their presence, Lambl's excrescences rarely cause valve regurgitation, therefore this diagnosis is not supported by our case.^7^

Infectious neoplasms are those caused by bacteria, fungi, or other microorganisms. Platelets, fibrin, red blood cells, white blood cells, and pathogens are the most prevalent pathological changes in the heart valve. Echocardiography facilitates the diagnosis by revealing rough‐edged and shaped growths on one or more leaflets. Given the patient's negative blood culture and normal CBC, the infectious neoplasm was not a suitable diagnosis.^8^

Typically, patients with rheumatic endocarditis, antiphospholipid syndrome, extramedullary proliferative disease, and solid tumors have noninfectious neoplasms. Autoimmune diseases manifest as thickened valves or tendons, with or without valve dysfunction. However, the medical history of our patient and the normality of inflammatory factors did not support the diagnosis of a noninfectious neoplasm.^1^

Most patients with severe AR will need aortic valve replacement (AVR). An aortic valve repair is only an option for patients whose anatomy is suitable (aortic dilation without a thickened, deformed, or calcified valve). AVR is, therefore, the primary treatment for AR caused by ACTS.^1^

Only 19 cases of ACTS have been reported since 1984, 15 of which were in men and four in women. The average age of reported cases was 55.85 ± 17.58 years, and the majority of patients presented with dyspnea. TEE revealed a single bicuspid aortic valve; additionally, TCVS was connected to RCC in five cases, LCC in five cases, NCC in six cases, and all cusps in four cases. AR occurred in 10 patients due to TCVS rupture (Table 1).

**TABLE 1:** Summary of case reports on ACTS.

First Author(ref)	Year	Race	Age (years), sex	Type of AV	Location of ASTS	Raptured cusp	AR	Sign and symptom	Treatment (Type of valve)
R Hashimoto^9^	1984	Japanese	10, male	Tricuspid	RCC	None	Not reported	Asymptomatic	AVR (Mechanical valve)
S Yavuz^10^	1999	Turk	46, male	Tricuspid	NCC	None	Severe	Not reported	AVR (Mechanical valve)
M Nakajima^11^	2002	Japanese	52, male	Tricuspid	all cusps	RCC	Severe	Not reported	AVR (Mechanical valve)
T Mishima^12^	2010	Japanese	18, male	Bicuspid	all cusps	None	Severe	Fatigue	AVR (Mechanical valve)
H Minami^13^	2011	Japanese	63, female	Tricuspid	NCC	RCC	Severe	Fatigue	AVR (Bioprosthetic valve)
K Akasaka^14^	2012	French	74, male	Tricuspid	all cusps	None	Severe	Dyspnea	AVR (Bioprosthetic valve)
AA Bouchachi^2^	2012	Japanese	56, female	Tricuspid	LCC	LCC	Severe	Not reported	AVR (Mechanical valve)
A Ishige^15^	2012	Japanese	56, female	Tricuspid	LCC	LCC	Severe	Heart failure	AVR (Mechanical valve)
Y Irisawa^4^	2014	Japanese	76, male	Tricuspid	all cusps	LCC	Severe	Dyspnea	AVR (Bioprosthetic valve)
I Esteve‐Ruiz^6^	2015	Spanish	70, male	Tricuspid	LCC	None	Severe	Not reported	Not reported
MM Abdelaziz^16^	2016	Englishman	66, male	Tricuspid	RCC	RCC	Severe	Not reported	CABG
S Ogawa^17^	2016	Japanese	67, male	Tricuspid	NCC	NCC	Moderate	Chest and back pain	AVR (Mechanical valve)
S Matsukuma^18^	2017	Japanese	60, male	Tricuspid	LCC	None	Severe	Chest pain	Aortic root remodeling
H Nishida^19^	2018	Japanese	53, male	Tricuspid	RCC	RCC	Severe	Dyspnea	AVR (Mechanical valve)
D Geindreau^20^	2018	United Kingdom	35, male	Not reported	NCC	None	Moderate	Not	Not reported
S Yuan^1^	2021	Chinese	70, male	Tricuspid	RCC&NCC	RCC	Severe	Dyspnea and chest tightness	AVR (Bioprosthetic valve)
X Wei^21^	2021	Chinese	56, female	Tricuspid	LCC	None	Severe	Dyspnea and chest pain	AVR
B Ophuis^22^	2021	Netherlands	73, male	Tricuspid	RCC	RCC	Severe	Circulatory shock	AVR (Mechanical valve)
Our case	2022	Iran	63, male	Tricuspid	NCC	None	Severe	Dyspnea	AVR (Mechanical valve)

## AUTHOR CONTRIBUTIONS

**Nakisa Khansari:** Conceptualization; data curation; formal analysis; investigation; methodology. **Amir Mohammad Salehi:** Conceptualization; formal analysis; funding acquisition; investigation; methodology.

## FUNDING INFORMATION

This research received no specific funding from any public, commercial, or not‐for‐profit funding agency.

## CONFLICT OF INTEREST STATEMENT

The author declares no conflict of interest.

## ETHICS STATEMENT

This study was approved by the Ethics Committee of Hamadan University of Medical Science.

## CONSENT

The patient has given his written permission for the publication of this report and the accompanying images. A manuscript of the informed consent form is available by contacting the corresponding author. This study was conducted in accordance with the Declaration of Helsinki.

## Supporting information


**Data S1:** Patient’s echocardiography video

## Data Availability

Access to data is permitted with the author's permission.
